# Study protocol: a core outcome set for perioperative exercise clinical effectiveness trials for lung cancer patients

**DOI:** 10.1186/s13063-024-07985-2

**Published:** 2024-03-01

**Authors:** Wanjun Zhou, Yawen Zhang, Zhiwei Wang, Liang Zhang, Xinqiong Zhang

**Affiliations:** 1 School of Nursing, Anhui Medical University, Hefei, 230032 People’s Republic of China; 2 The First Affiliated Hospital of Anhui Medical University, Hefei, 230032 People’s Republic of China

**Keywords:** Lung cancer, Perioperative exercise, Core outcome set, Outcome assessment, Protocol

## Abstract

**Background:**

Outcome assessment in perioperative exercise trials for lung cancer is heterogeneous, often omitting those that are important and patient-relevant. This heterogeneity hinders the synthesis of evidence. To address this issue, a core outcome set, an agreed-upon standardized set of outcomes to be measured and reported, is required to reduce heterogeneity among outcome measurements. This study protocol describes the methodology, aiming to develop a core outcome set for perioperative exercise intervention trials for lung cancer in clinical practice.

**Methods:**

The project will follow the standard methodology recommended by the Core Outcome Measures in Effectiveness Trials (COMET) initiative, which is divided into four steps. Stage I: Conducting a scoping review of outcomes reported in clinical trials and protocols to develop a list of potential outcome domains. Stage II: Conducting semi-structured interviews to obtain important outcomes for patients. Stage III: Choosing the most important outcomes by conducting two rounds of the Delphi exercise. Stage IV: Achieving a consensus in a face-to-face meeting to discuss the final core outcome set.

**Discussion:**

This is the first project identified for the core outcome set of perioperative exercise trials in lung cancer, which will enhance the quality, comparability, and usability of future trials and positively impact perioperative exercise and the care of patients with lung cancer.

**Trials registration:**

Core Outcome Measurement in Effectiveness Trials (COMET) Initiative database registration: https://www.comet-initiative.org/Studies/Details/2091

**Supplementary Information:**

The online version contains supplementary material available at 10.1186/s13063-024-07985-2.

## Introduction

### Background

Lung cancer (LC) is the second most common tumor worldwide, accounting for 11.4% of all new cancer cases and 18% of all cancer mortality across 36 cancers in 185 countries [1]. Surgical resection, as the effective radical treatment of early-stage LC [2], has increased the five-year survival rates of patients to 60% [3]. Although surgery offers a chance of cure, it also immediately impairs the cardiopulmonary function. Furthermore, the incidence of postoperative complications is increased, affecting the prognosis and potentially endangering the patient’s life [3]. Perioperative exercise has been shown to promote the rehabilitation of patients [4–6].

Exercise training, a subset of planned, structured, and repetitive physical activity that aims to improve or maintain physical fitness [7]. has been identified as an economical, safe, and effective treatment for lung cancer patients [8]. Currently, the number of clinical trials investigating the effectiveness of perioperative exercise in lung cancer patients is growing, which can provide conclusive evidence regarding the safety and efficacy of this intervention [9]. The selection and use of relevant, comparable, well-defined, and patient-important outcomes are crucial, impacting the trials’ validity, accuracy, and clinical applicability [10]. Commonly measured outcomes used to evaluate perioperative exercise include exercise capacity, lung function, postoperative complications, anxiety, breathlessness, respiratory muscle strength, and HRQoL [3, 4, 11]. Researchers often choose clinical trial outcomes that are easier to measure and require fewer resources, overlooking those important to patients and other stakeholders [12], which can lead to potential bias [9].

Particularly in trials evaluating perioperative exercise in LC, outcomes are heterogeneous due to the varied designs and implementation of the trials. While conducting a literature review, we found that outcome assessment, outcome reporting, and outcome definition exhibit heterogeneity in exercise intervention trials for lung cancer patients. More than 16 results were contained in the respiratory, thoracic, and mediastinal outcomes domain, while safety outcomes (adverse events).

and health economics outcomes (hospitalization costs and length) were assessed in less than a third of the studies. This hinders the comparison of trials and evidence synthesis (e.g., meta-analysis). The core outcome set (COS) is increasingly recognized to address the heterogeneity of outcomes and to help ensure that the most important outcomes are consistently assessed [13]. A COS is an agreed standardized set of outcomes that should be measured and reported in clinical trials, aiming to facilitate the synthesis of evidence and improve the consistency of reported outcomes [14].

Efforts are underway to develop a COS for pulmonary rehabilitation in lung cancer patients, specifically for post-discharge home-based or non-surgical treatments [15]. While there will likely be similarities in outcomes of importance for people with pulmonary rehabilitation and perioperative exercise, differences between the interventions and times mean different outcomes, Which includes a set of core outcomes for perioperative exercise with lung cancer is also need. This study aims to develop a core outcome set of clinically relevant perioperative exercise outcomes to promote the homogeneity of clinical trials, increase the availability and comparability of trials, and generate high-quality evidence.

### Aims and objectives

A core outcome set comprises the minimum agreed outcomes to be measured and reported in trials for a given health condition. Therefore, establishing what should be measured most during perioperative exercise in lung cancer patients is vital. The study aims to develop a core outcome set in collaboration with patients, health professionals, and researchers to examine the benefits of perioperative exercise for adults with LC. The main objectives are:

1. To identify a list of outcomes currently reported in perioperative exercise trials for LC.
2. To assess consistency in outcome reporting in published trials and protocols.
3. To explore the essential outcomes for stakeholders, including patients, healthcare professionals, researchers, and methodologists.
4. To achieve a consensus among multi-stakeholders on essential outcomes in this COS.

### Steering group

To oversee the development of the COS, we established an expert steering committee group composed of healthcare professionals, researchers, patients, and methodologists to guide the study’s design, recruitment, and development.

### Patient or public contribution

Patient and public representatives will be involved throughout the study to obtain multi-stakeholder perspectives on perioperative exercise outcomes.

## Method/design

**Fig. 1:**
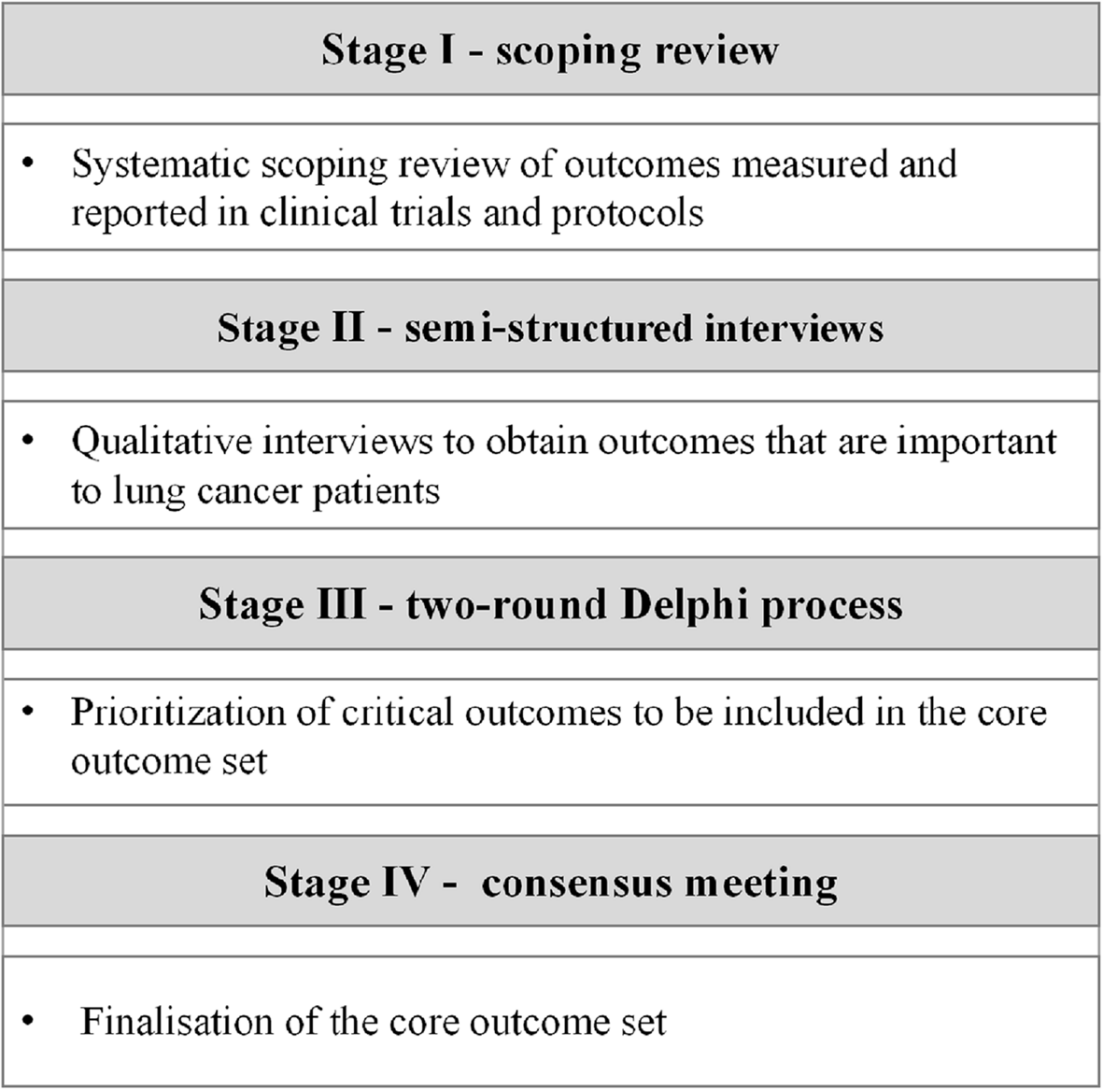
Flow diagram illustrating the process of developing the COS

The Core Outcome Measures in Effectiveness Trials (COMET) aims to establish a transparent methodology for COS. For the development and reporting of this COS, we will follow the rigorous process by COMET initiative, including the COMET Handbook [14] (Table 1), Core Outcome Set-STAndards Protocol Items (COS-STAP) [16], Core Outcome Set-STAndards for Development (COS-STAD) [17], Core Outcome Set-STAndards for Reporting (COS-STAR) [18], which have been successfully implemented in several high-quality COS projects [19, 20]. We will use a multi-stage approach combining qualitative and quantitative methods to develop the study protocol. Figure 1 provides an overview of the COS development steps.

- Stage I: Conducting a scoping review of outcomes reported in clinical trials and protocols to develop a list of potential outcome domains.
- Stage II: Conducting semi-structured interviews to obtain important outcomes for patients.
- Stage III: Choosing the most important outcomes by conducting two-round Delphi surveys.
- Stage IV: Achieving a consensus in a face-to-face meeting to discuss the final core outcome set.

**Table 1:**
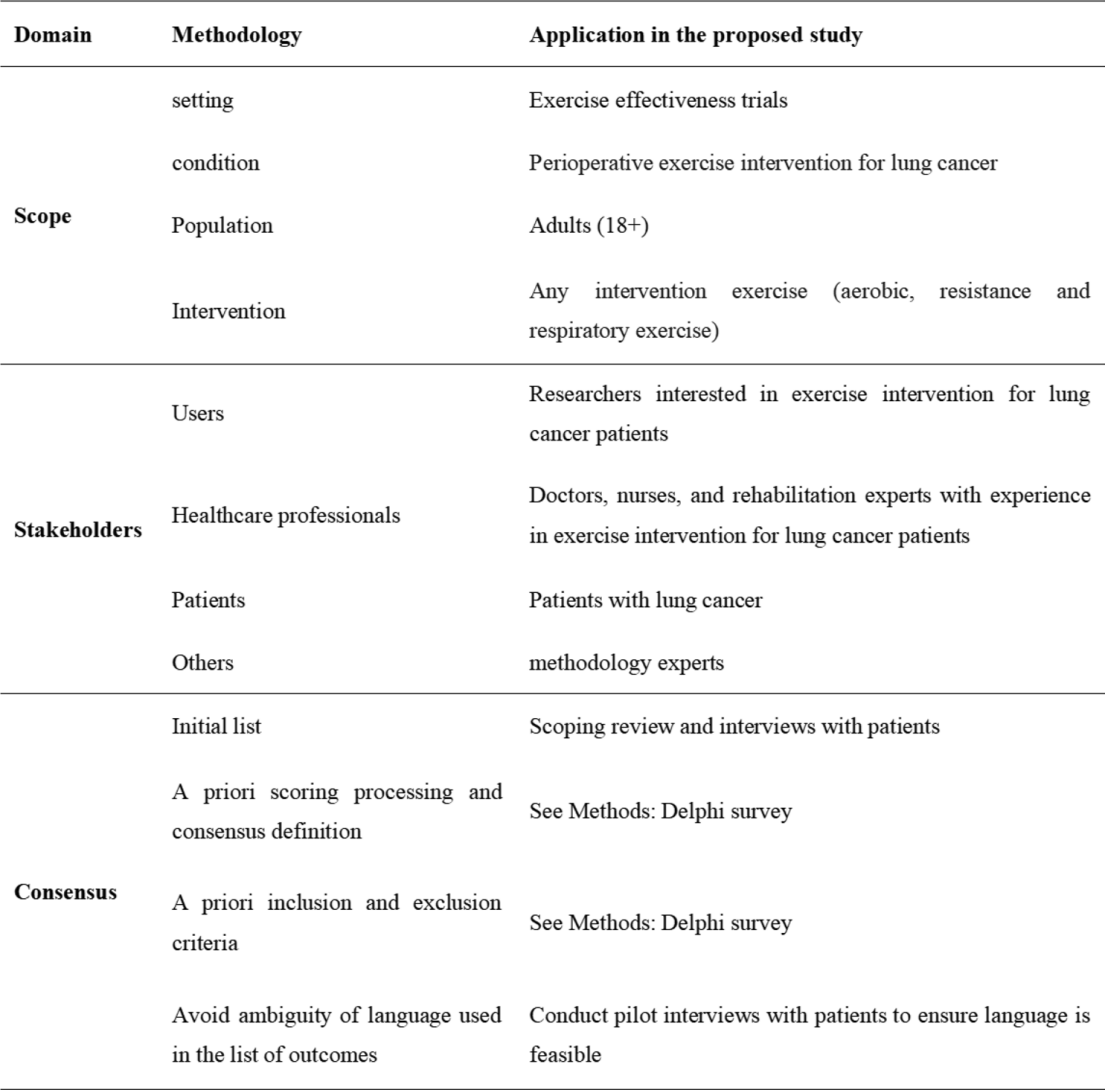
Developing COS process based on the COMET COS-STAD

### Stage I: a systematic scoping review of outcomes measured and reported for perioperative exercise with lung cancer

What outcomes are measured and reported in studies of perioperative exercise with lung cancer?

#### Search strategy

We will conduct systematic searches in the following electronic databases: PubMed, Web of Science, EMBASE, CNKI, WanFang database, VIP, CBM, Chinese Clinical Trials Registry, and ClinicalTrials.gov. Only quantitative studies will be included, while qualitative and mixed-method studies will be excluded.

#### Types of studies

We will include randomized controlled trials and quasi-randomized controlled trials on perioperative exercise interventions for lung cancer.

#### Types of interventions

We will include any intervention exercise encompassing aerobic, resistance, and respiratory training.

#### Types of participants

We will include lung cancer patients who have experienced perioperative exercise.

#### Exclusion criteria

1. Duplicated articles;
2. Lack of full text in Chinese or English or the inability to access the entire text; and
3. Chinese literature published in noncore journals and English literature published in non-SCI journals.

#### Data extraction

Articles will be excluded if their titles are not relevant. Two reviewers will independently review the search results. In case of discrepancies, a third reviewer will be consulted. Two reviewers will independently extract basic information about the study (first author, publication date, publication journal, literature title, and study type), sample information (sample size), intervention details (intervention duration, intervention type), and outcome information (outcome name, measurement time point, and measurement tool).

### Stage II: complementing the outcomes of perioperative exercise intervention in patients with LC

What outcomes do patients consider potentially important when undertaking perioperative exercise for LC?

#### Participant eligibility and sampling

Considering patients’ perspectives is essential in developing a COS. During the interviews, we will gather information about patients’ exercise experience, expectations, and relevant outcomes to understand their attitudes better. Samples will include adult patients diagnosed with lung cancer and previously treated by perioperative exercise intervention. Patient interviews will be conducted in China. We approached patients in the thoracic surgery ward at Anhui Medical University First Affiliated Hospital. Before conducting the interview, the patients will be asked to sign a written consent form indicating that they have been fully informed about the purpose of the interview and have given their consent to participate. Based on the literature review and clinical practice, the following semi-structured interview questions have been developed for patients [21] (Table 2).

**Table 2:**
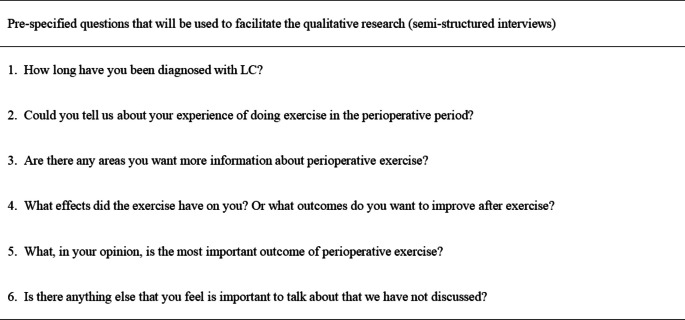
Pre-selected open-ended questions

There is no strict standard for the sample size of semi-structured interviews, and we will recruit 15 patients until the interview data reaches saturation [22]. To ensure the diversity of interview data, we will select patients of different genders, ages, disease stages, education levels, and occupations for interviews. Patients may withdraw at any time during the interview if they feel uncomfortable.

#### Data collected

The interviews will be audio-recorded, encompassing open-ended questions about the patients’ exercise experiences, desired exercise outcomes, and the impact on the patients.

#### Data analysis

Once transcribed and anonymized, the interview data will be thematically analyzed using NVivo software for data management. We will categorize the outcomes according to the COMET framework [23]. The Consolidated Criteria for Reporting Qualitative Research (COREQ) will be used to report the outcome of this qualitative study [24].

#### Review of outcome list

The scoping review and interviews will generate a list of outcomes for the Delphi exercise. We will determine the importance of these outcomes for patients based on their frequency. Utilizing the COMET taxonomy, we will group the outcomes into distinct domains. Patient representatives will be responsible for preparing and verifying a definition for each outcome in simple language.

The final survey will undergo testing to ensure clarity and feasibility before data collection. Before the first Delphi surveys, we will conduct a pilot test involving three types of stakeholders through email (3 patients, 3 healthcare professionals, and 2 researchers). Respondents will be asked to complete the questionnaire within 2 weeks of receiving the email. After evaluating and adjusting the questionnaire, we will proceed with 2 rounds of Delphi surveys. Respondents will be asked to complete the questionnaire within 2 weeks of receiving the email. After the questionnaire is evaluated and adjusted, two rounds of Delphi surveys will be conducted.

### Stage III: delphi survey

Delphi survey is a widely agreed upon method to develop consensus, which utilizes a process of sequential questionnaire completion and feedback to establish expert consensus between a panel of experts [25, 26]. A modified Delphi approach proposed by the COMET initiative involving two Delphi rounds will be used in this study [27].

#### Recruitment

Four types of stakeholders will be invited to participate in the survey to reflect the perspectives of experts in the field of perioperative exercise in lung cancer:

1. Patients with perioperative lung cancer who have received have experienced exercise;
2. Health professionals caring for LC patients (e.g., doctors, nurses, rehabilitation therapists);
3. Researchers (who care for patients but are also involved in designing research studies); and
4. Other stakeholders (methodology experts).

#### Sampling

There has yet to be a consensus for the optimal sample size for the Delphi, ranging from 12 to 174 for health professionals in previous studies [14]. Decisions about the number of participants to include in the Delphi process are pragmatic rather than based on statistical power. For the Delphi survey, we will attempt to invite every eligible participant, with 70 stakeholders expected to be invited to account for a 20% dropout rate. Participants will receive emails to complete the online Delphi survey within 2 weeks. To explain, we will provide personalized questionnaires and minimum waiting times between rounds 1 and 2.

#### Data collected

In the first round, the piloted candidate outcomes and associated description text will be presented. The order of the outcomes presented to participants will be randomly generated for each participant to eliminate the possibility of bias in the order of questions [28].

Stakeholders will be invited to rate the importance and suggest other outcomes that they consider important. In the Delphi exercise, a 9-point Likert scale will be used to rate the importance of each item (i.e., 1–3, not important; 4–6, important but not critical; 7–9, important and critical) [14]. If participants find it challenging to assign importance scores, they can also choose “not sure” for each result. An open-ended question will be included at the end of the questionnaire: Which outcomes do you think are important but not included? At this stage, the panel will review the new outcomes to decide whether to include them in the second round.

All outcomes from round one will be carried over to round two. Participants’ scores will be calculated for each outcome, and the results will be represented in a histogram based on the responses of the stakeholder groups. The steering committee will review additional outcomes suggested by stakeholders, and the new outcomes that gained the consent of the experts will appear in the second round of the Delphi exercise.

In the second round, we will invite the participants who completed the first round to participate again. Summary scores from all participants will be presented in the second round of the questionnaire. Participants will be invited to reflect on the feedback from the stakeholder group, re-score the outcomes, and score the additional outcomes suggested by participants in round one. In addition, if they change the rating, they will have the opportunity to explain their reasons.

#### Missing data

To reduce the loss of participants between Delphi survey rounds, we will send emails reminding participants to complete the Delphi survey by the end of the second week’s weekend. If the response rate is less than 80%, we will extend the opening time of the Delphi survey and invite other eligible individuals to participate.

The data will be lost if the participant’s questionnaire is not fully completed. We will attempt to contact the questioner to supplement the missing data. If we cannot reach them and there is only minimal data loss, we can utilize the previously filled-in data instead.

#### Consensus definition

Applying standardized consensus definitions to identify core outcomes: (1) Consensus: If ≥ 70% of respondents score 7–9 points, and ≤ 15% of respondents score 1–3 points, the outcome will be included in the final COS. (2) No consensus reached: If ≥ 70% of respondents score 1–3 points, and ≤ 15% of respondents rate 7–9 points, the outcome will be excluded in the final COS. The experts will discuss outcomes not reached after the Delphi survey at the consensus meeting.

### Stage IV: consensus meeting

This meeting may be held in person or virtually based on participants’ preferences and situations. If we can not hold a face-to-face consensus meeting, we will conduct an online meeting using a password-protected video conferencing platform. For this consensus meeting, we will invite healthcare professionals, researchers, methodologists, and patients who have participated in two rounds of the Delphi exercise. These key stakeholders not only have insight into the outcomes of perioperative exercise for patients with LC but are also potential users of the COS.

After 2 rounds of e-Delphi exercise, the results of each outcome score will be presented to all stakeholder groups. All participants will use the same scoring mechanism as the Delphi exercise. Stakeholder groups prioritize outcomes with consensus and discuss remaining issues sequentially. If the final COS is not reached at the end of the first consensus meeting, subsequent meetings will be considered.

## Discussion

Perioperative exercise plays an essential role in the rehabilitation of patients with LC. Increasing research has shown that perioperative exercise can reduce the adverse effects of surgery and improve lung function, exercise capacity, survival rate, and overall health [4, 11]. However, there is a pronounced heterogeneity and low comparability between studies. This makes it difficult to compare and pool results, restricting the application and promotion of perioperative exercise and hindering evidence synthesis [29]. How effective these exercise interventions are can only be truly understood if clinical trials report the same outcomes, which are measured and defined in the same way.

COS is crucial in the synthesis and transformation of evidence. The development of this study will follow the best methodological guidelines provided by the COMET initiative, standardizing the selection of clinical research outcomes and enhancing the value of clinical research. The COS will help improve the standardization of reporting relevant research outcomes and reduce heterogeneity in reporting similar studies.

The program will be constructed through a scoping review, semi-structured interviews, Delphi surveys, and consensus meetings to adopt multiple stakeholders’ views, ensuring the feasibility and promotion of this COS in future clinical trials. The development of COS provides consistent reporting of perioperative exercise research results for lung cancer patients, helping to reduce reporting bias. In the future, different clinical trial results can be compared and analyzed to improve the value of clinical research and reduce research waste.

### Dissemination

The COS will be disseminated through international publications and presented at relevant conferences to promote the implementation of this study. The study will be reported following COS-STAP, which is designed to reduce reporting bias and heterogeneity in the development process. In addition, this study aims to improve the quality of perioperative exercise clinical trials in lung cancer patients and facilitate the generation of high-quality evidence for systematic reviews/META analysis.

## Trials status

The outcome selection of the study has been completed and commenced in January 2022. Experts have been identified in the first Delphi round, which started on August 10, 2023. The intended completion will be May 1, 2024. This protocol is version 1.0, registered in July 2022.

### Supplementary Information

**Supplementary Material 1.**

## Data Availability

The datasets used and analyzed in this study are available upon request from the corresponding author.

## Abbreviations

LC: Lung Cancer
COMET: Core Outcome Measures in Effectiveness Trials
COS: Core Outcome Set
COS-STAP: Core Outcome Set-STAndards Protocol Items
COS-STAD: Core Outcome Set-STAndards for Development
COS-STAR: Core Outcome Set-STAndards for Reporting
